# Fine Mapping of Clubroot Resistance Loci *CRA8.1* and Candidate Gene Analysis in Chinese Cabbage (*Brassica rapa* L.)

**DOI:** 10.3389/fpls.2022.898108

**Published:** 2022-05-06

**Authors:** Yanyan Wang, Xianyu Xiang, Fan Huang, Wenlin Yu, Xueqing Zhou, Baojun Li, Yunyun Zhang, Peng Chen, Chunyu Zhang

**Affiliations:** ^1^College of Plant Science and Technology, Huazhong Agricultural University, Wuhan, China; ^2^Hybrid Rape Research Center of Shaanxi Province, Shaanxi Rapeseed Branch of National Centre for Oil Crops Genetic Improvement, Yangling, China; ^3^Industrial Crops Research Institute, Yunnan Academy of Agricultural Sciences, Kunming, China

**Keywords:** clubroot, *Plasmodiophora brassicae*, *Brassica rapa*, *CRA8.1*, fine mapping

## Abstract

Clubroot is caused by *Plasmodiophora brassicae*, which threatens *Brassicaceae* crop production worldwide. In recent years, there has been an outbreak and rapid spread of clubroot in many major cruciferous crop-producing areas of China. In this study, we identified a cabbage material DingWen (DW) with different resistant capabilities from Huashuang5R (H5R) and Huayouza62R of *Brassica napus*, which are currently used as the main resistant cultivars for clubroot management in China. We used a next-generation sequencing-based bulked segregant analysis approach, combined with genetic mapping to identify clubroot-resistant (CR) genes from F_1_ population generated from a cross between the DW (CR) and HZSX (clubroot susceptible). The CR locus of DW (named *CRA8.1*) was mapped to a region between markers A08-4346 and A08-4853, which contains two different loci *CRA8.1a* and *CRA8.1b* after fine mapping. The *CRA8.1b* loci contain a fragment of 395 kb between markers A08-4624 and A08-4853 on A08 chromosome, and it is responsible for the resistance to *PbZj* and *PbXm* isolates. However, together with *CRA8.1a*, corresponding to a 765-kb region between markers A08-4346 and A08-4624, then it can confer resistance to *PbXm*^+^. Finally, through expression analysis between resistant and susceptible materials, two genes encoding TIR-NBS-LRR proteins (*BraA08g039211E* and *BraA08g039212E*) and one gene encoding an RLP protein (*BraA08g039193E*) were identified to be the most likely CR candidates for the peculiar resistance in DW.

## Introduction

Clubroot is a highly contagious soil-borne disease that specifically endangers *Brassica* species, and it is caused by *Plasmodiophora brassicae*, a pathogen that belongs to peculiar eukaryotic taxa under Endomyxa branch (Dixon, 2009). Most of the Brassica plants, including *Brassica napus*, *Brassica oleracea*, and *Brassica rapa*, can be infected by *P. brassicae.* The susceptible plants display a swollen root symptom by this disease infection, which leads to dysfunction of root function for water and nutrient transport, eventually withering and death (Gahatraj et al., 2019). In recent years, the disease is rapidly spreading from different continents and countries, resulting in severe yield loss of cruciferous crops and vegetables (Hwang et al., 2012).

Although biological and chemical reagents can rescue the disease phenotype under certain conditions, the identification and introgression of resistance genes to create resistant varieties is the most efficient and economic approach to defeat the disease spreading (Elke et al., 2009; Chai et al., 2014). A few qualitative and quantitative clubroot-resistant (CR) loci have been identified in *B. rapa*, *B. oleracea*, *B. napus*, and *Brassica nigra* (Mehraj et al., 2020). The majority of CR genes are located on A genomes, including A01, A02, A03, A05, A06, and A08 chromosomes (Mehraj et al., 2020). In particular, CR loci on A03 or A08 chromosomes are extensively studied, including *PbBa3.1*, *PbBa3.2*, and *PbBa3.3*, *CRd*, *Crr3*, *CRk*, *CRb*, *Rcr4*, *Rcr1*, and *CRa* from A03 chromosome (Matsumoto et al., 1998; Hirai et al., 2004; Piao et al., 2004; Sakamoto et al., 2008; Chen et al., 2013; Chu et al., 2014; Yu et al., 2017; Pang et al., 2018), and *Crr1* (including both *Crr1a* and *Crr1b*), *PbBa8.1*, *Rcr3, Rcr9wa*, *BraA.CR.b*, and *Rcr9* from A08 chromosome (Suwabe et al., 2003; Chen et al., 2013; Yu et al., 2017; Hirani et al., 2018; Karim et al., 2020). *CrrA5* and *Crr4* were located on A05 and A06 chromosomes (Suwabe et al., 2006; Nguyen et al., 2018), and *Crr2, PbBa1.1*, *CRc*, and *Rcr8* were mapped to A01 and A02 chromosomes, respectively (Suwabe et al., 2003; Sakamoto et al., 2008; Chen et al., 2013; Yu et al., 2017). Based on fine-mapping markers, some CR loci reside extremely close to or overlap each other, such as *Crr1* and *PbBa8.1*, and *CRa* and *CRb*, and they can be allelic and actually originate from homologous genes from different A-genome species (Chen et al., 2013). These genes can be used as resources for breeding CR accessions.

Actually, from a broader view of disease resistance genes, most plant resistance (R) genes identified so far encode for cell membrane or intracellular receptors, including RLPs/RLKs (receptor-like proteins/kinases) and NLRs (nucleotide-binding site–leucine-rich repeat) (Kourelis and van der Hoorn, 2018). Plant innate immunity system comprises PTI (PAMP-triggered immunity) and ETI (effector-triggered immunity) (Deng et al., 2020). It is believed that specific interactions between a resistance gene and certain pathotypes are dependent mainly *via* ETI, but also affected by PTI pathway (Kim et al., 2016; Neik et al., 2017; Larkan et al., 2020; Yuan et al., 2021). For PTI, PAMPs from the invading pathogen are perceived by host membrane receptors known as PRRs (pattern recognition receptors), and RLKs and RLPs are the two major groups of PRRs on the cell surface determining the initial recognition (Wu and Zhou, 2013; Larkan et al., 2015; Grund et al., 2019; Kim et al., 2020). For the ETI route, effectors released inside the host cell are recognized by plant intracellular proteins such as NLRs, some of which require additional helper NLRs or chaperon proteins for triggering the ETI response (Grund et al., 2019). Different types of NLR proteins have been identified based on their protein domain structure, such as TIR-NBS-LRR, CC-NBS-LRR, and CC_*R*_-NBS-LRR subgroups (Wang and Chai, 2020). The above-mentioned CR genes, only two CR genes, namely, *CRa* and *Crr1a*, have been successfully isolated, both encoding TIR-NBS-LRR (NLRs) proteins (Ueno et al., 2012; Hatakeyama et al., 2013).

Clubroot-resistant genes from *B. rapa* have been introgressed into susceptible *B. napus* in order to generate resistant varieties. Currently, the two CR *B. napus* varieties in China are Huashuang5R (H5R) and Hayouza62R, which were generated through crosses using resistant *B. rapa* donor parents ECD04 (AA, 2n = 20) and Shinki (AA, 2n = 20), respectively (Zhan et al., 2020; Li et al., 2021). They were resistant to most of the clubroot pathogens isolated from different regions in China (Nadil et al., 2019). However, due to the soil-borne nature of the clubroot pathogen, a “resistant” Brassica variety to one pathogen can become “susceptible” to another pathogen from a different source. The present clubroot pathogen classification systems include the “Williams” system (Williams, 1966), the ECD system (Buczacki et al., 1975), and the SCD system (Pang et al., 2020). According to the “Williams” system, pathotype 4 is the most prevalent form in China, and most of the isolates such as *PbZj* (Zhijiang isolated from Hubei province) and *PbXm* (Xinmin from Liaoning province) belong to this group. The two *B. napus* CR varieties H5R and Hayouza62R containing *PbBa8.1-* and *CRb*-resistant loci, respectively, are resistant to *PbZj* and *PbCd* (Chengdu) isolates, but not to *PbXm* pathogen (Shah et al., 2020). Another struggling problem is the loss of resistance over time, which also calls for a new resource of resistance genes. During previous work, people found that pyramiding multiple CR genes in one variety would greatly improve the resistance of the host plant. For example, *CRb* and *PbBa8.1* pyramiding lines in *B. napus* have demonstrated that plants in a homozygous state in each CR gene/locus had higher resistance than those in a heterozygous state (Shah et al., 2020). However, the fast spreading of clubroot disease, the presence of various pathotypes for the clubroot pathogens from different regions, and the frequent loss of resistance collectively call for urgent identification of a new CR gene resource.

The purpose of this study was to identify a CR locus different from *PbBa8.1* and *CRb* and to identify the candidate genes responsible for disease resistance, therefore providing foundation for future utilization of these new CR genes for resistance breeding in *Brassica* species.

## Materials and Methods

### Plant Materials

The Brassica cultivars used in the current study include H5R, Huashuang5S (H5S), and 409R (the resistant restore line for Huayouza62R) of *B. napus*, DingWen (DW), HuangZiShaXun (HZSX, Z1) (Belser et al., 2018), and 91-12 of *B. rapa*. H5R and 409R are resistant cultivars containing CR loci of *PbBa8.1* and *CRb*, respectively. H5S and 91-12 were used as susceptible controls. DW is a hybrid and sterile resistant material. A susceptible cultivar HZSX was used as a parent in crosses with DW for F_1_ population construction and gene mapping.

### *Plasmodiophora brassicae* Collection and Clubroot Resistance Phenotyping

Different species of *P. brassicae* from the Chinese cabbage roots with severe galling were collected from five different locations of China: *PbXm*, Xinmin of Liaoning province; *PbCd*, Chengdu of Sichuan province; *PbZj*, Zhijang of Hubei province; *PbTc*, Tengchong of Yunnan province; and *PbLx*, Linxiang of Yunnan province. During the study, we found that Xinmin region contained two isolates, based on the host response. Therefore, we named it as “*PbXm*^+^,” in order to discriminate it from the other one. Galls were frozen and stored in −20°C until further use.

Clubroot resistance test of all materials was carried out as described previously (Chen et al., 2015). In brief, the collected galls were thawed at room temperature, ground to homogenization with buffer, and filtered through gauze, and resting spores were isolated after rounds of centrifugation. The spore concentration was measured and diluted to 10^7^ resting spores per milliliter in sterile distilled water, before being used for host plant inoculation.

Host plants were prepared by germination of seeds on moist filter paper for 4 days, followed by the growth of seedling in a nursery room on medium at 16-h photoperiod, with 25/20°C day/night temperature for 7 days. When seedlings are ready, 1 ml of the above resting spore suspension was inoculated into the root of the seedling, and the disease symptoms were scored after 30–40 days. Clubroot severity was scored as 0–3 based on the root morphology, with grade 0 as normal growth, grade 1 as a few small galls on the lateral roots, grade 2 as big galls on lateral roots, and grade 3 as big galls on both primary roots and lateral roots. The disease index (DI) was calculated based on the number of plants at various severity levels (Shah et al., 2020).

### Bulked Pool Construction and Sequencing

Bulked segregation analysis (BSA) method was used to map the genomic region responsible for CR in DW. A genetic cross was performed using DW and HZSX, and the two parents were genetically variant and also showed contrasting CR phenotype. Thirty-eight highly resistant and 38 extremely susceptible plants were selected from F_1_ population to construct two pools (resistant pool and susceptible pool), respectively. Genomic DNA was extracted from leaves of individual plants using DNA Secure Plant Kit (TIANGEN, China). The DNA concentration was quantified with a NanoDrop 2000 spectrophotometer (Thermo Fisher Scientific, United States), and DNA of each individual plant within the same pool is mixed in equal concentration, resulting in two DNA libraries for sequencing.

Second-generation sequencing was performed on these two libraries using an Illumina HiSeq platform in PE150 mode. The raw data were accessed for quality control and then transformed into clean data by removing adaptor sequence. Clean reads were mapped to the reference genome of *B. rapa*^1^ using the Burrows–Wheeler Aligner (BWA0.7.12-r1039 mem).

### *CRA8.1* Gene Mapping

To identify candidate CR loci in DW, we first calculated the Euclidean distance (ED) value of SNP and InDel separately and then constructed maps based on the distribution of ED value on the chromosome. The molecular markers, developed by Zhan et al. (2020) on the A08 chromosome, were applied for genotyping. Five hundred F_1_ plants were used in the primary mapping, and the population was expanded to 3290 F_1_ plants for fine mapping. Physical map was constructed according to the location of molecular markers using MapChart software (Voorrips, 2002).

### Assembly of *CRA8.1* Fragment

To get sequence information of fragment containing *CRA8.1* in DW, we performed fragment assembly with a European turnip ECD04 genome as the reference.

First, based on a Nova Seq 6000 sequencing platform, around 100 Gb Illumina short reads were obtained with leaf DNA of disease-resistant individual R59, which is the resistant plant in F_1_. Then, the Illumina short reads were mapped to the reference genome by BWA software package and rearranged by Samtools software package with parameters all set as default (Li and Durbin, 2009). Second, SNP/InDels data were extracted from the mapping results by BCF tools software package with the parameters set as “DP > 30 and QUAL > 100” (Narasimhan et al., 2016). At the same time, based on the sequence of A08-4346 and A08-5076 markers and blast result from NCBI^2^ from the reference genome, chromosomal fragments between the two markers from the reference genome were extracted by bedtools software package with default parameters (Quinlan and Hall, 2010).

Then, based on SNP/InDels data, an R script was written to replace the nucleotide sequence of candidate fragment of DW. The non-heterozygous SNP/InDels loci were replaced directly, while for loci containing heterozygous SNP/InDels, only one of the SNP/InDels was randomly selected to replace the candidate sequence.

### Gene Synteny Analysis of *CRA8.1* Fragment

First, we downloaded the Chinese cabbage HuangZiShaXun (Z1) genome,^3^ *B. napus* ZS11 genome,^4^ and European turnip ECD04 genome (unpublished). Then, the CDS of each genome was extracted by GFF read software package with parameter set as default. Finally, the gene synteny analysis was performed with CDS of candidate fragment as query and CDS of each genome as subject with MCscan.^5^

### Identification and Expression Analysis of Candidate Genes

We used DW and HZSX (susceptible parent) and inoculated them with *PbZj* or *PbXm*^+^ isolates, respectively. Root samples were taken at 0 h, 12 h, and 4 days after inoculation, total RNA was extracted, and the candidate gene expression was analyzed using qRT-PCR. Information of qRT-PCR primers is listed in Supplementary Table 1, and *BrUBC10* (XM_009134237.3) was used as an internal reference.

For promoter *cis*-element analysis, the DNA sequence 3.0 kb upstream of the coding sequence was submitted to the Plant CARE database^6^ for the prediction of putative *cis*-elements. The data were presented using TBtools software.

## Results

### Chinese Cabbage “DW” With Broad Resistance to *Plasmodiophora brassicae* From Different Regions

**FIGURE 1:**
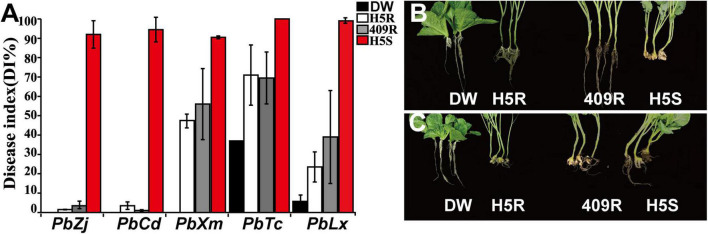
Disease resistance responses of different materials to *P. brassicae* from different field isolates. **(A)** Disease index statistics. Black, white, gray, and red bars represent the disease index in DW, Huashuang5R (H5R), 409R, and Huashuang5S (H5S), respectively. **(B,C)** The phenotypes of DW, H5R, 409R, and H5S were inoculated with *PbZj* and *PbXm*, respectively.

Previously, a Chinese cabbage material was screened out with excellent resistance to Zj isolate of *P. brassicae*, and this material was named “DW.” In order to further verify the pathogen resistance profile of DW and characterize the identity of CR genes in comparison with the ones currently widely used in H5R and Huayouza62R *B. napus* varieties in China, DW, H5R, 409R (resistant, restore line of Huayouza62R), and H5S were inoculated by five different clubroot isolates collected from different locations of China, namely, *PbXm*, *PbCd*, *PbZj*, *PbTc*, and *PbLx* (Figure 1A). Indoor inoculation experiments showed that susceptible control H5S was severely infected by all pathogens, while H5R, 409R, and DW had variable resistance (Figure 1A). H5R, 409R, and DW showed high resistance to *PbZj* and *PbCd* (DI ≤ 3.5%) (Figures 1A,B), and DW was the only one resistant to *PbXm*, whereas H5R and 409R showed a DI level of 47.5 and 56%, respectively (Figures 1A,C). We can also see that all four materials used in this study were susceptible to *PbTc*; however, DW displayed lower DI than H5R and 409R (Figure 1A). Similarly, H5R and 409R showed 24–40% DI to *PbLx*, but the DI of DW was below 10% (Figure 1A). Taken together, these results suggest that DW is a material with superior CR, which is fundamentally different from that in H5R and 409R, suggesting possible different genetic background difference behind their resistance.

### Fine Mapping Revealed at Least Two Resistance Loci Presented in DingWen

**FIGURE 2:**
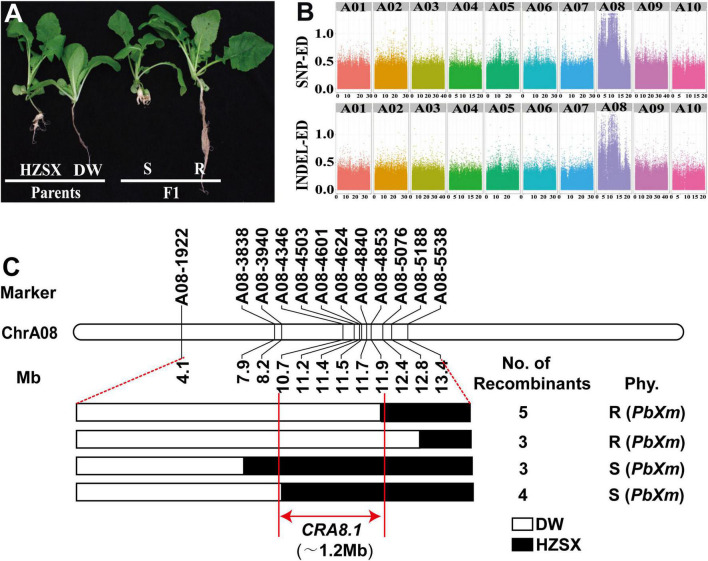
Primary mapping of *CRA8.1* in *B. rapa*. **(A)** Root phenotypes of DW, HZSX, and F_1_ after inoculation with *PbXm*. S, susceptible; R, resistant. **(B)** The Euclidean distance (ED) values of SNPs and InDels on chromosomes from the A genome. **(C)** Fine mapping of *CRA8.1* region with InDel markers. Fifteen recombinants were identified from F_1_ population from markers A08-1922 to A08-5538. Black or white represents the regions found in HZSX or DW, respectively. Red lines indicate the *CRA8.1* locus (1.2 Mb) identified from this study.

To investigate the DW inheritance of the resistance, a segregating population was constructed using a susceptible material HZSX crossed with DW. Parental lines (DW and HZSX) and 139 F_1_ individuals were subsequently inoculated with the *PbXm* and *scored* for their resistance. As shown in Table 1, DW showed complete resistance with DI of 0, whereas HZSX was completely susceptible with DI of 100% (Table 1). The F_1_ plants from a cross of DW with HZSX exhibited roughly 1:1 segregation (among 139 F_1_ individuals, 69 and 70 were resistant and susceptible, respectively), indicating that the CR loci in DW are controlled by a single dominant gene (Figure 2A and Table 1).

**TABLE 1:** Disease phenotype statistics in F_1_ population from cross of DW and HZSX.

Parents and crosses	Type	Disease levels				Total	IR^a^	χ ^2^
		0	1	2	3			
DW	R-parent	30	0	0	0	30	0	
HZSX	S-parent	0	0	0	30	30	100	
DW × HZSX	F_1_	69	0	0	70	139	50	0.007

*^a^Incidence rate (%).*

In order to identify the resistance loci, BSA (bulked segregation analysis) pools consisting of R- and S-plants were used for high-throughput sequencing. A total of 278,237,232 and 271,955,410 raw reads were obtained from R- and S-pools, respectively (Supplementary Table 2). After removing the adaptor sequence and filtering off low-quality reads, we recovered over 93% clean reads from the R- and S-pools, respectively, with Q30 over 87% (Supplementary Table 2). The clean reads were mapped to *B. rapa* reference genome (V3.0), and the total mapping rates were over 96%.

According to the ED of SNP and InDel (Figure 2B), a region of about 15 Mb was identified on A08 chromosome associating with CR (Figure 2B). To narrow down the region in genome, 12 molecule markers from the developed molecular markers were screened, and 15 recombinants were screened between markers A08-1922 and A08-5538 in F_1_ population. They were divided into four groups carrying different chromosomal fragments based on genotyping results (Figure 2C). Based on the resistance phenotype and the genotyping results, the CR loci were primarily mapped between A08-4346 and A08-4853 intervals, corresponding to a region of 1.2 Mb on chromosome A08 of *B. rapa* genome (V3.0) (Figure 2C). Interestingly, a previously known resistance locus *PbBa8.1* overlaps with this region (Zhan et al., 2020). Since DW was highly resistant to *PbXm* pathogen, while H5R was not, this is a new locus conferring different CR profiles from *PbBa8.1*, and we named this locus as *CRA8.1* hereafter.

**FIGURE 3:**
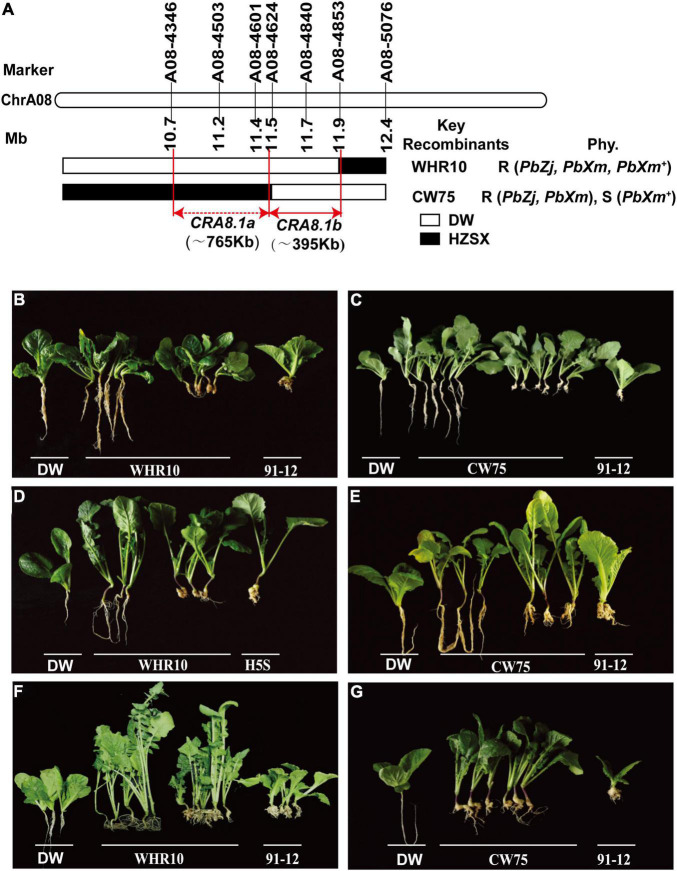
*CRA8.1a* or *CRA8.1b* loci identified by fine mapping from A08-4346 to A08-4853 markers. **(A)** Genotyping of two representative recombinants CW75 and WHR10, carrying *CRA8.1b* or *CRA8.1a* and *CRA8.1b*, respectively. Disease phenotype of WHR10 and CW75 progeny plants after inoculation of *PbZj* **(B,C)**, *PbXm* **(D,E)**, or *PbXm*^+^ **(F,G)**, respectively.

To further narrow down the region of *CRA8.1* and eventually identify the candidate genes, we expanded the F_1_ population for fine mapping. Finally, 39 recombinants were obtained from 3290 F_1_ plants. Two different recombinant individuals, namely, WHR10 and CW75, were obtained, and they were sterile. We backcrossed them with HZSX, and the progenies were inoculated by *PbZj*, *PbXm*, and *PbXm*^+^, together with DW and Chinese cabbage 91-12, as positive and negative controls, respectively (Figure 3). When *PbXm* or *PbZj* was used, the backcross progeny of these two recombinants with HZSX also showed 1:1 segregation (Figures 3B–E and Supplementary Table 3); however, when *PbXm*^+^ was used, the offspring of WHR10 showed a segregation ratio of 1:1, while those from CW75 were all susceptible (Figures 3F,G and Supplementary Table 3). Based on the BSA sequencing, the fragment between A08-4346 and A08-4624 intervals, in a total length of 765 kb, might be responsible for the resistance phenotype variation between CW75 and WHR10 for *PbXm*^+^ (Figure 3A). Therefore, we concluded that *CRA8.1* contains at least two disease-resistant loci, conferring resistance to different isolates (*PbXm*^+^ and *PbXm*/*PbZj*). The candidate region named as *CRA8.1b* covers a region of 395 kb between markers A08-4624 and A08-4853, and it contributes to resistance to *PbZj*/*PbXm* isolates. The other locus *CRA8.1a*, corresponding to a region between A08-4346 and A08-4624, may confer resistance to *PbXm*^+^ by itself or coordinately with *CRA8.1b* (Figure 3A).

### Three Candidate Clubroot-Resistant Genes Identified in *CRA8.1* Region Based on Resequencing and Sequence Comparison

**FIGURE 4:**
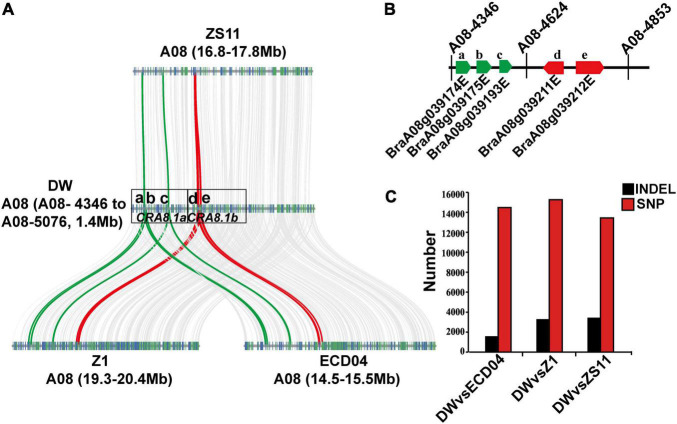
Genome structure comparison between homologous regions on A08 chromosome from DW, HZSX (Z1), ECD04, and ZS11. **(A)** Gene synteny analysis between chromosomal regions from A08-4346 to A08-5076 on A08 chromosome from DW, ECD04, ZS11, and Z1. a–e represent five candidate R genes found within this region. a–c belongs to the *CRA8.1a* region. d, e belongs to the *CRA8.1b* region. **(B)** Genomic orientation of the five candidate R genes between A08-4346 and A08-4853 on A08 chromosome. Green and red bars represent the candidate genes in *CRA8.1a* and *CRA8.1b* loci, respectively. **(C)** Number of SNPs and InDels in DW compared with ECD04, Z1, and ZS11 between the target regions flanked by markers of A08-4346 and A08-5076.

In order to identify candidate CR genes in the region between A08-4346 and A08-4853 intervals on A08 chromosome, we resequenced one of the resistant recombinant individuals R59 generated from the F_1_ population. The sequencing yielded 422 million clean reads with 63 billion clean bases. We manually assembled this region using ECD04 as the reference genome (unpublished) and compared gene distribution between DW, HZSX, and, a widely used *B. napus* variety, ZS11 (Figure 4). The total length of this assembly was 1.4 Mb (including the fragment between markers A08-4346 and A08-5076), and we found a good collinearity between DW, Z1 (HZSX), ZS11, and ECD04 (Figure 4A). Pairwise comparison within this segment suggested there were on average level of 14,395 SNPs and 2776 InDels that were different in DW to ZS11, ECD04, or Z1 varieties (Figure 4C).

It is reported that 61% of the cloned R genes code for NLRs and 19% of R genes for RLPs/RLKs (Kourelis and van der Hoorn, 2018). Based on NLR/RLP annotation, we identified five R genes within the candidate region of A08 chromosome, corresponding to the following gene sequences of ECD04: *CRA8.1.1* (*BraA08g039174E*), *CRA8.1.2* (*BraA08g039175E*), *CRA8.1.3* (*BraA08g039193E*), *CRA8.1.4* (*BraA08g039211E*), and *CRA8.1.5* (*BraA08g039212E*) (Figure 4A and Table 2). Among those candidate genes, the homologs of *BraA08g039174E*, *BraA08g039175E*, and *BraA08g039193E* are typical RLP genes and homologs of *BraA08g039211E* and *BraA08g039212E* are TIR-NBS-LRR genes (Figure 4B and Table 2). Homologs of *BraA08g039174E*, *BraA08g039175E*, and *BraA08g039193E* are located in *CRA8.1a* region, while the remaining two are in *CRA8.1b* region (Figure 4A).

**TABLE 2:** List of CR candidate genes identified within *CRA8.1* loci.

Gene ID (ECD04)	AGI (Arabidopsis gene)	Gene type
BraA08g039174E	AT3G05360.1	RLP
BraA08g039175E	AT1G71400.1	RLP
BraA08g039193E	AT3G05360.1	RLP
BraA08g039211E	AT4G19500.1	TIR-NBS-LRR
BraA08g039212E	AT4G19510.1	TIR-NBS-LRR

We reason that if an R gene is present in both resistant and susceptible plants, the difference may reside on gene sequence (including CDS and promoter) or expression level, or both. Since DW is a heterozygous material for resistance, in order to find out the resistant allelic genes in DW, we designed primers covering 3.0 kb upstream and the whole CDS region using information of genome resequencing (Supplementary Table 1). After T-A cloning and sequencing, genes with sequence variation between DW and HZSX were considered as genes possibly responsible for the new type of CR in DW.

Then, in order to clarify whether there is a functional diversification between resistant versus susceptible varieties, we extracted gene homologs from ECD04, ZS11, SL (Sheng Li), and QU (Quinta),^7^ the latter three as susceptible *B. napus* representatives. We compared gene coding sequences using BioEdit software (Supplementary Figures 1–5). We found that the protein sequences of *BraA08g039174E* and *BraA08g039175E*, both as LRR-domain-only proteins, were similar between different accessions (Supplementary Figures 1, 2). As for the third LRR-domain-only protein, *BraA08g039193E*, the DW and ECD04 protein sequences were quite different compared with the ones in ZS11, SL, and QU materials (Supplementary Figure 3). The protein sequence of *BraA08g039211E* in DW showed two missing amino acids (S223, Y224 in ECD04, and Z1) and one substitution (E191) in TIR domain; otherwise, the sequences were quite similar (Supplementary Figure 4). In the case of *BraA08g039212E*, the sequence in DW was almost identical to that of ECD04, but different from that in susceptible Zl, ZS11, and other accessions (Supplementary Figure 5).

**FIGURE 5:**
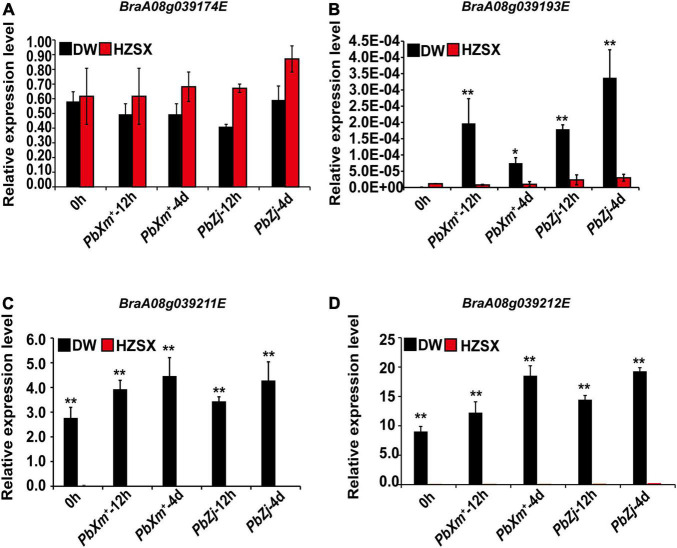
Relative expression of candidate CR genes between DW (black) and HZSX (red) before and after inoculation of *PbXm*^+^ and *PbZj* isolates. **(A–D)** Relative expression of homologs of *BraA08g039174E, BraA08g039193E*, *BraA08g039211E*, and *BraA08g039212E*, respectively. ** represents *p* ≤ 0.01 by Student’s *t*-test.

We also quantified the relative expression of these candidate genes in DW or Z1 (HZSX) upon inoculation of *PbXm*^+^ or *PbZj*, except for *BraA08g039175E* whose expression was too low (Figure 5). As shown in Figure 5, the expression of *BraA08g039174E* was quite similar between DW and HZSX, both before and after inoculation (Figure 5A). On the contrary, we observed a clear difference on the expression levels of *BraA08g039211E* and *BraA08g039212E* between DW and HZSX, in particular an obvious induction on *BraA08g039193E* in DW upon infection (Figures 5B–D). Since these genes are NBS-LRR or RLP genes and also show differential expression between susceptible and resistant materials responding to pathogen infection, they are considered as the final candidate R genes awaiting further functional verification.

**FIGURE 6:**
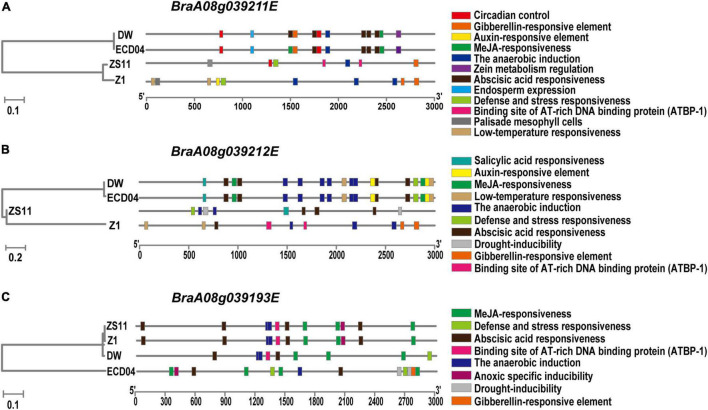
Analysis of *cis*-acting elements in promoter region of the homologs of *BraA08g039211E* **(A)**, *BraA08g039212E* **(B)**, and *BraA08g039193E* **(C)** between resistant (DW and ECD04) and susceptible (ZS11 and Z1/HZSX) materials.

In order to find the reason for different transcript levels between resistant and susceptible materials, especially in DW, we extracted promoter sequence and predicted regulatory *cis*-elements within these regions from DW, ECD04, ZS11, and Z1(HZSX) (Figure 6). As shown in Figure 6, similar patterns were observed in promoter regions of *BraA08g039193E*, *BraA08g039211E*, and *BraA08g039212E* from ECD04 and their corresponding orthologs in DW, but different from that in susceptible ZS11 and Z1 (Figure 6). For example, the MeJA-responsive elements in the promoter regions of *BraA08g039211E* and *BraA08g039212E* were very similar in DW and ECD04, but not compared to Z1 and ZS11 (Figures 6A,B). In the case of *BraA08g039193E*, the *cis*-elements in ZS11 and Z1 were similar, followed by DW, but with additional defense- and stress-responsive elements. The *cis*-element in ECD04 was different from the remaining three, and whether this is of any biological relevance needs further verification (Figure 6C).

Taken together, our data from the expression profile, protein sequence, and promoter *cis*-element analysis suggest that *BraA08g039211E* and *BraA08g039212E* from *CRA8.1b* region are the most likely candidates leading to resistance to *PbZj*/*PbXm*. In addition, *BraA08g039193E* is likely to be the main candidate gene in *CRA8.1a*, but *BraA08g039211E* and *BraA08g039212E* may interact with *BraA08g039193E* to resist *PbXm*^+^ isolate coordinately.

## Discussion

In this study, we discovered a unique *B. rapa* material DW that showed different resistance profiles other than H5R (*PbBa8.1*) or Huayouza 62R (*CRb*), suggesting possible new CR genes involved in its resistance. Fine mapping narrowed down to a genomic region with two NBS-LRR genes and one RLP gene as potential candidates. Once validated for their function as CR genes, those candidate genes could confer broader and higher resistance to more virulent clubroot pathogens. Therefore, genes from DW provide a new gene resource for CR breeding in future.

### *CRA8.1* Is Different From *PbBa8.1* and Confers Superior Resistance to Diverse Pathogens

Numerous CR loci have been identified previously from ECD04 and other *B. rapa* materials, for instance, *PbBa8.1* and *Crr1a* on A08 chromosome (Chen et al., 2013; Hatakeyama et al., 2013) (Table 3). In this study, our locus *CRA8.1* is also located on chromosome A08 in DW, and genetically, this locus is dominant based on the F_1_ population segregation results. *PbBa8.1* is the source of resistance in H5R; however, H5R was susceptible to *PbXm* pathogen, suggesting new genes in DW being responsible for its resistance. In the process of fine mapping, we found that there were two loci *CRA8.1a* and *CRA8.1b* in the primary localization interval of *CRA8.1*. Homologs of *BraA08g039211E* and *BraA08g039212E* are the most promising candidate genes in DW for *CRA8.1b*, which may play an important role against *PbZj* and *PbXm* isolates. However, what is the different resistance of *BraA08g039211E* and *BraA08g039212E* in DW and H5R remains to be further studied. The resistance of DW to *PbXm*^+^ comes from *CRA8.1a* itself or *CRA8.1a* and *CRA8.1b* together. So, it is not clear whether there is an interaction between the homologous genes of *BraA08g039211E* and *BraA08g039212E* and other resistance genes, or there are other types of resistance genes independently involved in the resistance response. In order to solve this problem, it is necessary to deepen the fine mapping of *CRA8.1* loci, which involves the screening and isolation of recombinant plants with *CRA8.1a* locus only and subsequent inoculation of its offspring with *PbXm*^+^ to observe the segregation ratio.

**TABLE 3:** Clubroot-resistant loci presently known in *Brassica rapa*.

Location	Source	Name of loci	Pathogen	Classification	References
A01	G004 (Siloga derived)	*Crr2*	Wakayama-01	Williams (race 4)	Suwabe et al., 2003
A01	ECD04	*PbBa1.1*	Pb2, Pb7	Williams (race 2, race 7)	Chen et al., 2013
A02	C9 (Debra derived)	*CRc*	K04	n.a.^a^	Sakamoto et al., 2008
A02	T19 (Pluto derived)	*Rcr8*	Pathotype 5x	Williams (race 5^b^)	Yu et al., 2017
A03	ECD04	*PbBa3.1*	Pb2	Williams (race 2)	Chen et al., 2013
A03	ECD04	*PbBa3.2*	Pb10	Williams (race 10)	Chen et al., 2013
A03	ECD04	*PbBa3.3*	Pb7	Williams (race 7)	Chen et al., 2013
A03	Chinese cabbage inbred line 85-74	*CRd*	Pb4	Williams (race 4)	Pang et al., 2018
A03	N-WMR-3 (Milan White derived)	*Crr3*	Ano-01	Williams (race 4)	Hirai et al., 2004
A03	K10 (CR Kanko derived)	*CRk*	M85 and K04	n.a.^a^	Sakamoto et al., 2008
A03	Chinese cabbage cultivar, CR Shinki	*CRb*	Pb4	Williams (race 4)	Piao et al., 2004
A03	T19 (Pluto derived)	*Rcr4*	Pathotypes 2, 3, 5, 6, and 8	Williams	Yu et al., 2017
A03	ECD1, ECD2, ECD4	*BraA.CR.a*	n.a.	n.a	Hirani et al., 2018
A03	ECD3	*BraA.CR.c*	n.a.	n.a	Hirani et al., 2018
A03	ECD02	*BraA3P5X.CRa/b ^Kato^1.1*	Pathotype 5x	Williams (race 5^b^)	Fredua-Agyeman et al., 2020
A03	ECD02	*BraA3P5X.CRa/b ^Kato^1.2*	Pathotype 5x	Williams (race 5^b^)	Fredua-Agyeman et al., 2020
A03	Pak choy cv. ‘Flower Nabana’	*Rcr1*	Pathotype 3	Williams (race 3)	Chu et al., 2014
A03	Chinese cabbage inbred line T 136-8	*CRa*	Pathotype 2	Williams (race 2)	Matsumoto et al., 1998
A03	Chinese cabbage cv. ‘Jazz’	*Rcr2*	Pathotype 3	Williams (race 3)	Huang et al., 2017
A06	G004 (Siloga derived)	*Crr4*	Wakayama-01, Ano-01	Williams (race 4)	Suwabe et al., 2003
A08	G004 (Siloga derived)	*Crr1*	Wakayama-01	Williams (race 4)	Suwabe et al., 2003
A08	ECD1, ECD2, ECD3, ECD4	*BraA.CR.b*	n.a.	n.a	Hirani et al., 2018
A08	ECD04	*PbBa8.1*	Pb4	Williams (race 4)	Chen et al., 2013
A08	96-6990-2 (Waaslander derived)	*Rcr3*	Pathotype 3H	Williams (race 3)	Karim et al., 2020
A08	T19 (Pluto derived)	*Rcr9*	Pathotype 5x	Williams (race 5^b^)	Yu et al., 2017
A08	ECD04	*Rcr9^Wa^*	Pathotype 5x	Williams (race 5^b^)	Karim et al., 2020

*^a^n.a., not available. ^b^Pathotype 5x is a new pathotype identified by Yu et al. (2017).*

### The New Type of Clubroot-Resistant Genes That Can Be Used for Revealing the Novel Resistance Mechanisms

Based on gene distribution around *CRA8.1* locus in ECD04, ZS11, DW and HZSX (Z1), the resistance spectrum of DW is unique and most likely coming from the *CRA8.1a* fragment (Figure 4). The candidate genes were the only NBS-LRR or RLP/RLK genes within this region, of which five can be quantified for relative expression. Among the three final candidates, *BraA08g039211E* and *BraA08g039212E* are actually located head to head adjacently on the chromosome, and both are drastically induced from 12 h up to 4 days in DW after clubroot infection (Figures 4B, 5C,D). Previous studies have shown that some of the R genes work in pair, and they are physically located very close on the chromosome, such as TNL pair genes RRS1/RPS4 from *Arabidopsis thaliana* (Le Roux et al., 2015) and CNL pair genes RGA5/RGA4 in rice (Cesari et al., 2013). So, *BraA08g039211E* and *BraA08g039212E* as TIR-NBS-LRR genes might be very important for host response to the clubroot pathogen. The third gene, *BraA08g039193E* is a RLP protein. This is distinct from the previously cloned NLR-type genes and mediates immune responses at the PTI level. Once the candidate gene is validated, what is the disease resistance mechanism is also a very interesting scientific question. In addition, *BraA08g039211E* has little residue difference between DW and HZSX, *BraA08g039193E* and *BraA08g039212E*, the sequence in DW was quite different compared with the ones in ZS11, SL, and QU materials, so they can be used as good materials for resistance mechanism research in future.

### DingWen Is an Excellent Clubroot-Resistant Donor Plant for Resistance Improvement of *Brassica napus*

As mentioned above, clubroot is a fast-spreading soil-borne disease which calls for fast and effective resistant varieties. The current resistance cultivars can revert to susceptible upon long terms of use, and single or a handful of CR genes cannot assure resistance to different pathotypes of the clubroot pathogens. Also, the accumulation of mutations eventually would result in the loss of resistance. For example, in Alberta of Canada, a “new” brassicae pathogenic type that can overcome resistance was discovered only 4 years after the CR variety release (Strelkov et al., 2016; Fredua-Agyeman et al., 2018). Most CR genes used currently are coming from *B. rapa*, since crossability between *B. rapa* and *B. napus* is very good, and CR genes from *B. rapa* can readily be transferred to *B. napus*. The current two widely used CR varieties of *B. napus*, namely, H5R and 409R, are resistant to *PbZj* but not to *PbTc* and *PbXm* (Shah et al., 2020). Previous studies have shown that different disease resistance genes’ aggregation can improve the resistance to clubroot (Suwabe et al., 2006; Shah et al., 2020). In this study, the *CRA8.1* locus located in DW contains at least two CR loci, which is superior to *PbBa8.1* and *CRb* on A08 chromosome, so the two resistance genes of *CRA8.1* can be introduced into excellent *B. napus* parents to improve the CR in breeding, which can save cost and improve work efficiency.

## Data Availability Statement

The original contributions presented in the study are included in the article/Supplementary Material, further inquiries can be directed to the corresponding authors.

## Author Contributions

YW performed the experiments, analyzed the data, prepared the figures, and drafted the manuscript. XX helped to analyze the data. CZ and PC conceived the study, participated in its coordination, and helped to draft the manuscript. All authors have read and approved the final manuscript.

## Conflict of Interest

The authors declare that the research was conducted in the absence of any commercial or financial relationships that could be construed as a potential conflict of interest.

## Publisher’s Note

All claims expressed in this article are solely those of the authors and do not necessarily represent those of their affiliated organizations, or those of the publisher, the editors and the reviewers. Any product that may be evaluated in this article, or claim that may be made by its manufacturer, is not guaranteed or endorsed by the publisher.

## Footnotes

1
http://39.100.233.196:82/download_genome/Brassica_Genome_data/Brara_Chiifu_V3.0/

2
https://blast.ncbi.nlm.nih.gov/Blast.cgi

3
http://www.genoscope.cns.fr/plants

4
http://cbi.hzau.edu.cn/bnapus/

5
https://github.com/tanghaibao/jcvi/wiki/MCscan-(Python-version)

6
http://bioinformatics.psb.ugent.be/webtools/plantcare/html/

7
http://yanglab.hzau.edu.cn/BnTIR

## Supplementary Material

Supplementary Figure 1 Protein sequence alignments of homologs of *BraA08g039174E* from DW, ECD04, Z1, ZS11, SL, and QU. Red solid lines represent the LRR domain.

Supplementary Figure 2 Protein sequence alignments of homologs of *BraA08g039175E* from DW, ECD04, Z1, ZS11, SL, and QU. Red solid lines represent the LRR domain.

Supplementary Figure 3 Protein sequence alignments of homologs of *BraA08g039193E* from DW, ECD04, Z1, ZS11, SL, and QU. Red solid lines represent the LRR domain.

Supplementary Figure 4 Protein sequence alignments of homologs of *BraA08g039211E* from DW, ECD04, Z1, ZS11, SL, and QU. Green solid lines represent the TIR domain, orange solid lines represent the NB-ARC domain, and red solid lines represent the LRR domain. The symbol “**” indicates the two missing residues in DW compared with other accessions. The symbol “*” represents the specific residues in DW.

Supplementary Figure 5 Protein sequence alignments of homologs of *BraA08g039212E* from DW, ECD04, Z1, ZS11, SL, and QU. Green solid lines represent the TIR domain, orange solid lines represent the NB-ARC domain, and red solid lines represent the LRR domain. The symbol “*” represents the specific residues in DW.

Supplementary Table 1 Primers used in this study.

Supplementary Table 2 Quality control of BSA sequencing data.

Supplementary Table 3 Phenotype statistics of clubroot resistance for progeny of WHR10 and CW75 recombinants.
