# Supplementary figures and images for: Fine Mapping of Clubroot Resistance Loci CRA8.1 and Candidate Gene Analysis in Chinese Cabbage (Brassica rapa L.)
Source: Front Plant Sci. 2022 May 6;13:898108. doi: 10.3389/fpls.2022.898108 (PMC9121064; doi:10.3389/fpls.2022.898108)

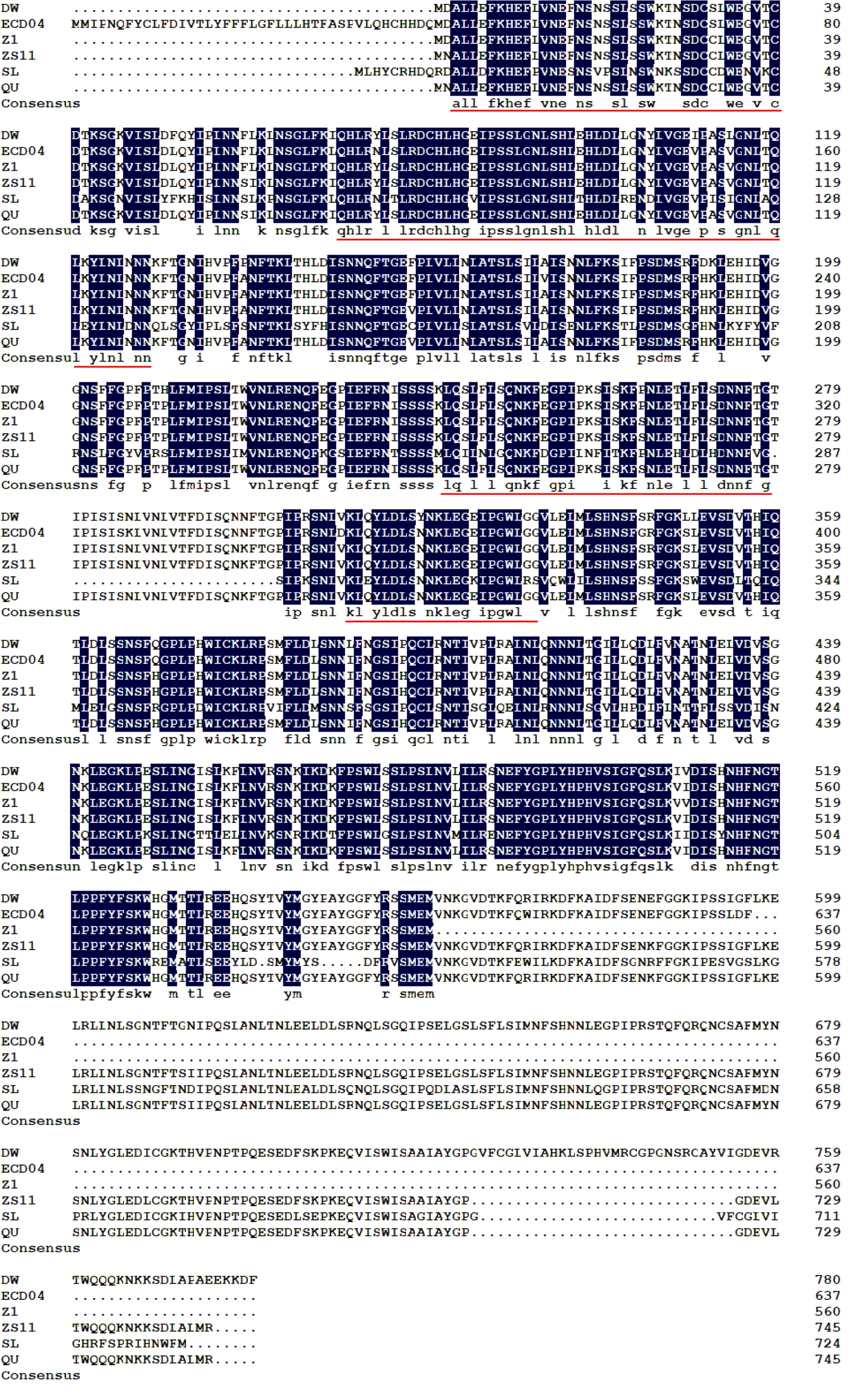

Supplement: Supplementary Figure 1 — Protein sequence alignments of homologs of BraA08g039174E from DW, ECD04, Z1, ZS11, SL, and QU. Red solid lines represent the LRR domain. [file Image_1.TIF]

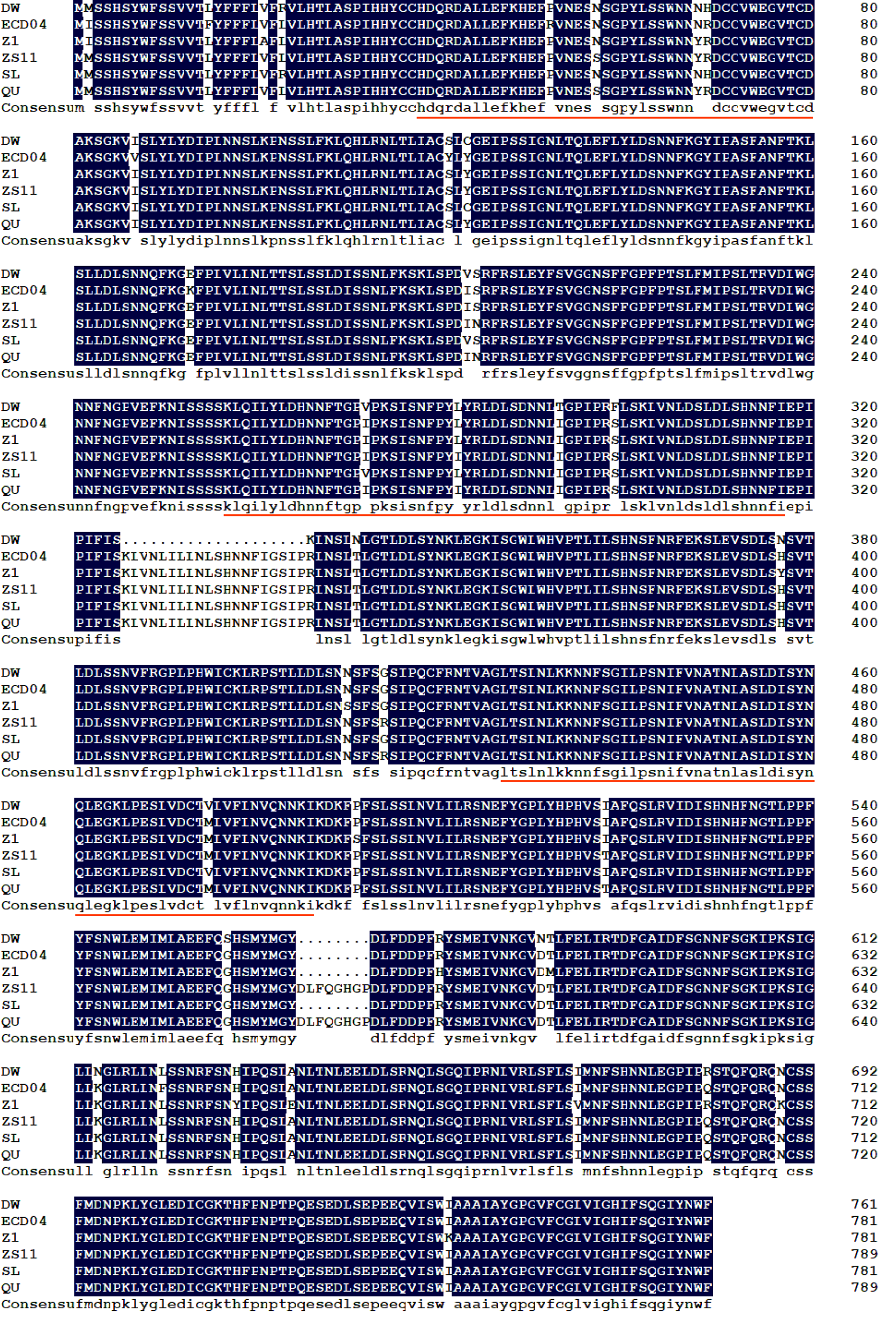

Supplement: Supplementary Figure 2 — Protein sequence alignments of homologs of BraA08g039175E from DW, ECD04, Z1, ZS11, SL, and QU. Red solid lines represent the LRR domain. [file Image_2.TIF]

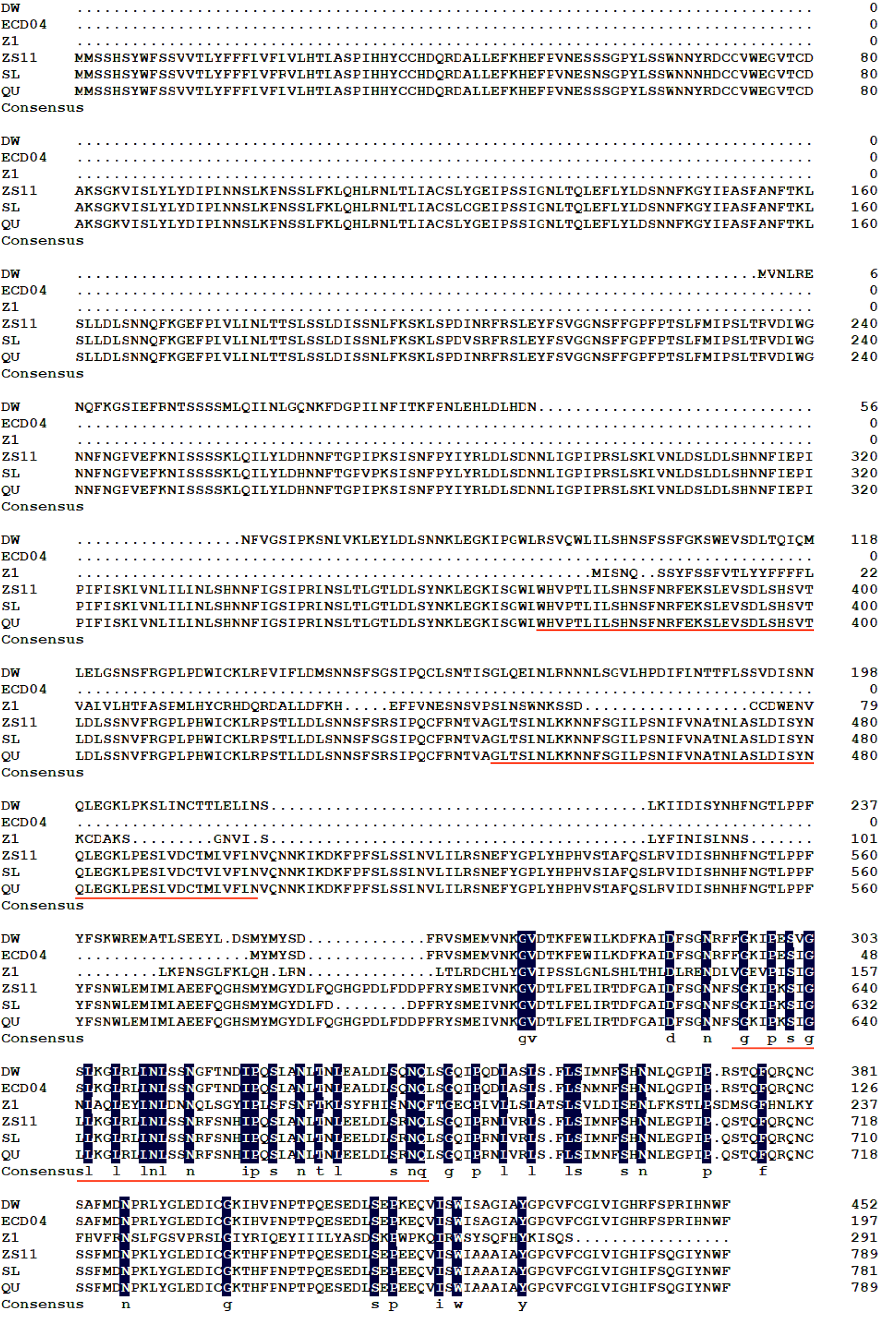

Supplement: Supplementary Figure 3 — Protein sequence alignments of homologs of BraA08g039193E from DW, ECD04, Z1, ZS11, SL, and QU. Red solid lines represent the LRR domain. [file Image_3.TIF]

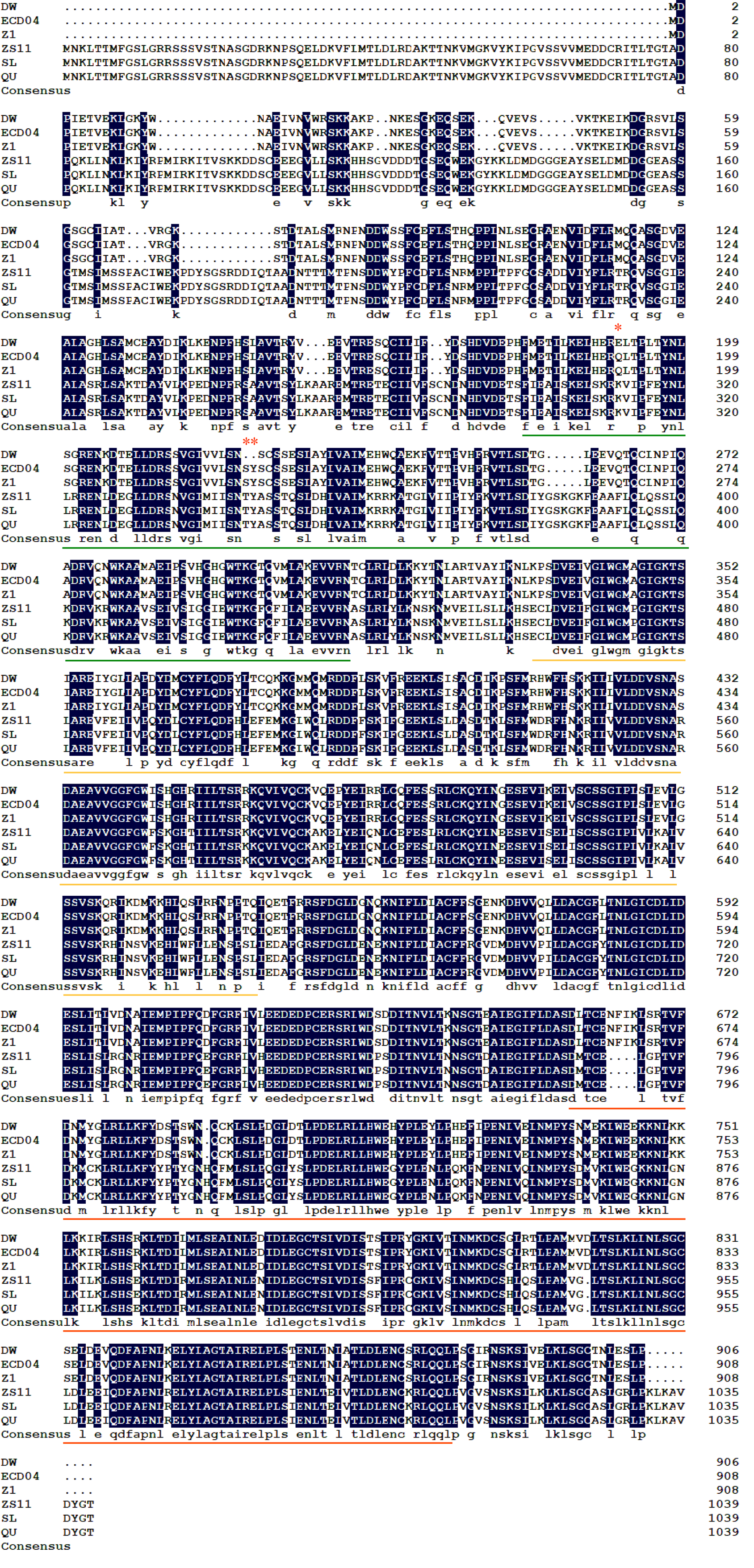

Supplement: Supplementary Figure 4 — Protein sequence alignments of homologs of BraA08g039211E from DW, ECD04, Z1, ZS11, SL, and QU. Green solid lines represent the TIR domain, orange solid lines represent the NB-ARC domain, and red solid lines represent the LRR domain. The symbol “**” indicates the two missing residues in DW compared with other accessions. The symbol “*” represents the specific residues in DW. [file Image_4.TIF]

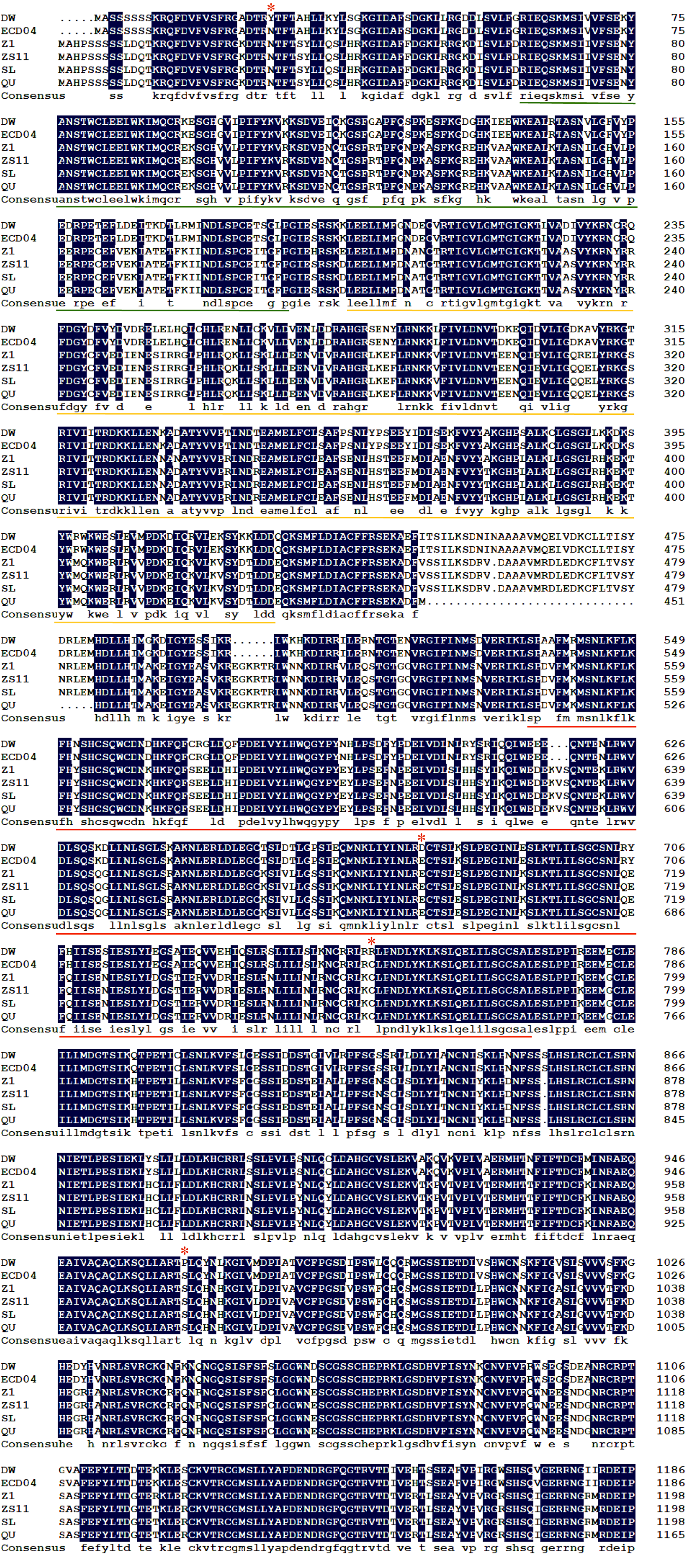

Supplement: Supplementary Figure 5 — Protein sequence alignments of homologs of BraA08g039212E from DW, ECD04, Z1, ZS11, SL, and QU. Green solid lines represent the TIR domain, orange solid lines represent the NB-ARC domain, and red solid lines represent the LRR domain. The symbol “*” represents the specific residues in DW. [file Image_5.TIF]
